# Adverse reactions to etiological treatment in patients with Chagas disease in the Western Potiguar mesoregion of Brazil: a cross-sectional study

**DOI:** 10.1590/1516-3180.2024.0154.R2.04112025

**Published:** 2026-01-09

**Authors:** Henrique Rangelly Gabriel de Melo, Aurélio Julio Silva Dantas, Maria Gabriela Augusto de Medeiros Jácome, Milena Sonely Mendonça Bezerra Lima, Antônia Suellen Fernandes Dantas, José Antonio da Silva, Ellany Gurgel Cosme do Nascimento, Cléber de Mesquita Andrade

**Affiliations:** I Universidade do Estado do Rio Grande do Norte (UERN), Mossoró (RN), Brazil.; II Universidade do Estado do Rio Grande do Norte (UERN), Mossoró (RN), Brazil.; III Universidade do Estado do Rio Grande do Norte (UERN), Mossoró (RN), Brazil.; IV Universidade do Estado do Rio Grande do Norte (UERN), Mossoró (RN), Brazil.; V Dietitian. Universidade do Estado do Rio Grande do Norte (UERN), Mossoró (RN), Brazil.; VI Registered Nurse. Universidade do Estado do Rio Grande do Norte (UERN), Mossoró (RN), Brazil.; VII Registered Nurse. Universidade do Estado do Rio Grande do Norte (UERN), Mossoró (RN), Brazil.; VIII Universidade do Estado do Rio Grande do Norte (UERN), Mossoró (RN), Brazil.

**Keywords:** Chagas disease, Drug-related side effects and adverse reactions, Drug therapy, Benznidazole, Etiological treatment, Prevention

## Abstract

**BACKGROUND:**

Chagas disease is a significant public health challenge in Brazil that is characterized by substantial morbidity and mortality rates, coupled with limited etiological treatment options.

**OBJECTIVE:**

To describe and assess the occurrence of adverse reactions resulting from benznidazole treatment in patients from the Western Potiguar mesoregion of Brazil.

**DESIGN AND SETTING:**

This retrospective, longitudinal, descriptive observational study included 106 individuals with Chagas disease who attended the Chagas Disease Outpatient Clinic of the Universidade do Estado do Rio Grande do Norte.

**RESULTS:**

Among patients subjected to etiological treatment with benznidazole, 40.5% (43/106) experienced adverse reactions, manifesting in 13 distinct forms. The most prevalent reactions occurred primarily in the dermatological and hematological systems. Thus, despite the notable frequency of adverse reactions, their severity remained low, as evidenced by the minimal treatment suspension rate. The treatment demonstrated potential benefits to those affected by the disease.

**CONCLUSIONS:**

This study characterized the most frequent adverse reactions to benznidazole, mainly dermatological and hematological reactions, which were mostly mild and rarely led to treatment suspension. Recognizing these events is essential for guiding professionals, enhancing patient confidence, and improving adherence to etiological treatments for Chagas disease.

## INTRODUCTION

 Chagas disease is caused by the *Trypanosoma cruzi* protozoan. Despite more than a century of research, it remains a significant public health concern in Brazil, particularly in socioeconomically vulnerable populations. In Latin American countries, approximately 15 genera of vectors can transmit the protozoan to humans, with the primary vectors being *Triatoma, Panstrongylus*, and *Rhodnius*, which typically inhabit peridomestic regions.^1,2^

 In the definition of the chronic phase, patients may present with different clinical forms. The indeterminate form is characterized by at least two reactive serologies using distinct methodologies, absence of characteristic cardiac and digestive symptoms of the disease, as well as normal electrocardiogram, chest radiography, and contrast studies of the esophagus and colon.^3^ The cardiac form is characterized by the onset of cardiac symptoms such as palpitations and dyspnea, electrocardiographic alterations such as intraventricular and atrioventricular conduction disturbances, and various ventricular and supraventricular arrhythmias. Patients with cardiac involvement may demonstrate alterations in segmental contractility on echocardiography, including apical or vorticular aneurysms of the left ventricle, or even biventricular involvement. The digestive form of the disease is characterized by the appearance of symptoms such as dysphagia of solids and liquids and/or constipation, resulting from the action of *T. cruzi* on the myenteric plexuses, potentially leading to the development of megacolon and/or megaesophagus.^4^

 A systematic literature review and meta-analysis has indicated that between 1980 and 2012, the average prevalence of Chagas disease in Brazil was 4.2%. The prevalence throughout this period was higher in the central-west (4.7%), northern (4.2%), southeast (4.1%), northeast (4%), and southern regions (2%) than in other regions. Bahia (20.4%), Pernambuco (9.1%), Paraíba (7.8%), and Rio Grande do Norte (5.9%), among other regions, had the highest prevalence.^5^

 Between 1978 and 1980, the northeastern region of Brazil presented a significant infection prevalence, accounting for 3.05% of the prevalence of Chagas disease; the overall prevalence in Brazil was 4.4%, with the most significant data recorded in Bahia, Alagoas, Sergipe, Pernambuco, and Piauí.^6^ In the 1960s, seroprevalence data in Rio Grande do Norte reached 15.5%.^7^ The Western Potiguar mesoregion included municipalities of relevance for seroprevalence in the state, with Severiano Melo, Felipe Guerra, Apodi, Caraúbas, Campo Grande, and Governador Dix-Sept Rosado being the notable areas. Together, they account for an approximate seroprevalence of 6.5% in the state, affecting approximately 14,000 individuals.^8,9^

 According to the Brazilian Society of Cardiology Guidelines on Diagnosis and Treatment of Chagas Disease Cardiopathy Patients, etiological treatment is strongly recommended for children with acute and congenital infections, adults and adolescents with acute or recently acquired infections, children and adolescents with chronic infections, and adults aged < 50 years with indeterminate chronic infections.^10^

 Currently, benznidazole (5 mg/kg/day) and nifurtimox (10 mg/kg/day) are used for etiological treatment, with a minimum chemotherapy duration of 60 days. Only benznidazole is available for the treatment of Chagas disease in Brazil.^11^

 The mechanism of action of benznidazole remains unclear. It likely acts as a prodrug with trypanocidal effects after undergoing action by type I trypanosomal nitroreductases, thereby becoming an active product owing to the action of these oxygen-insensitive enzymes expressed in protozoa. This action culminates in the blockade of new DNA strand synthesis and inhibition of *T. cruzi’s* rudimentary antioxidant system, making it susceptible to oxidative damage.^12,14^

 Etiological treatment of patients with Chagas disease with benznidazole has an efficacy of 97.9% in congenital infection (treatment performed between 0 and 6 months), 71.5% in the acute phase, 57.6% in the recent chronic phase, and 5.9% in the late chronic phase.^15^ Moreover, its adverse reaction rate is 38%, with the most common outcome being cutaneous rash.^16^

## OBJECTIVE

 This study aimed to evaluate and describe the occurrence of adverse reactions in patients with Chagas disease in an endemic area of northeastern Brazil who received etiological treatment with benznidazole. 

## METHODS

 This was an observational, descriptive, longitudinal, and retrospective study conducted on patients from the Chagas Disease Outpatient Clinic of the Universidade do Estado do Rio Grande do Norte (ADOC-UERN). The patients originated from endemic areas in the Western Potiguar mesoregion and received etiological treatment for Chagas disease. 

 Patients included in the study had reactive serologies for *T. cruzi* performed using at least two different methods (indirect immunofluorescence, enzyme-linked immunosorbent assay, or indirect hemagglutination) and were prescribed benznidazole at ADOCUERN. The study included adults in the acute or chronic phase of the disease, either in the indeterminate form or with mild involvement of the cardiac, digestive, or cardiodigestive forms. 

 The only exclusion criterion was prior etiological treatment before being followed up at ADOC-UERN, considering that the inclusion of these patients in the study would result in bias because of the inability to monitor and measure the effects presented by the patients before they were admitted to the service. 

 Patients received 5 mg/kg/day of benznidazole, divided into three doses, with the maximum dose being limited to 300 mg/day. For patients weighing > 60 kg, treatment was extended for another day for each kilogram of weight > 60 kg, not exceeding 80 days of treatment. Patients were followed up with clinical and laboratory evaluations, including complete blood count, aspartate aminotransferase and alanine aminotransferase activity, and creatinine concentration, before treatment initiation, every 15 and 30 days, and at the end of etiological treatment. 

 Adverse reactions were meticulously recorded in individual medical records according to complaints and physical examination findings during routine outpatient follow-ups, followed by immediate medical evaluation and appropriate intervention for each case. Of the 520 patients receiving care at ADOC-UERN, 106 were included after applying the aforementioned inclusion and exclusion criteria. 

 Data are described using absolute and relative frequencies. Bivariate analysis was conducted using Pearson’s chi-squared test and Fisher’s exact test, when appropriate. Significance was set P < 0.05, with 95% confidence intervals (CI). 

 Some risks of methodological biases were observed, including the difficulty of standardizing laboratories responsible for collecting and analyzing data from examinations and the challenge of facilitating access to patients to monitor laboratory results with more precision, given the diverse geographical distribution of individuals served at ADOC-UERN. To mitigate these biases, laboratory tests should be conducted in facilities that adhere to the respective municipal health departments, aiming for reliability of the results obtained. 

 Before inclusion in the care program of ADOC-UERN, all patients signed an informed consent form approved by the Research Ethics Committee of the Universidade do Estado do Rio Grande do Norte (1,160,553 and CAAE: 43783915.3.00005294, July 21, 2015). This study adhered to the ethical principles outlined in Resolution 466/12 of the National Health Council of Brazil for research involving humans. 

## RESULTS

 Of the total of 106 participants, aged between 18 and 65 years, 54.7% (58/106) were men, and 45.3% (48/106) were women. The most prevalent clinical form was the indeterminate form (69.8%, 74/106), followed by the cardiac (15.1%, 16/106), digestive (10.4%, 11/106), and cardiodigestive forms (4.7%, 5/106). 

 Among the patients who received etiological treatment with benznidazole, 40.5% (43/106) experienced adverse reactions, which were observed in 12 different manifestations and distributed in 55 events (more than one reaction could be observed in the same patient). The primary reactions observed were dermatitis (27.3%; 15/55) and pruritus (16.4%; 9/55) (**Table 1**). 

**Table 1:** Frequency of adverse reactions in patients with Chagas disease treated with benznidazole at the Chagas Disease Outpatient Clinic of the Universidade do Estado do Rio Grande do Norte (ADOC-UERN)

**Systems n (%)**	**Adverse reaction**	**Sex**		**Sex**
		**Male**	**Female**	
		**n (%)**	**n (%)**	**n (%)**
Dermatological 30 (54.5)	Pruritus	1 (5.3)	8 (22.2)	9 (16.4)
	Dermatitis	5 (26.3)	10 (27.8)	15 (27.3)
	Xerosis with peeling	2 (10.5)	4 (11.1)	6 (10.9)
Hematological 9 (16.4)	Leukopenia	4 (21)	2 (5.5)	6 (10.9)
	Thrombocytopenia	2 (10.5)	1 (2.8)	3 (5,4)
Gastrointestinal 8 (14.5)	Xerostomia	1 (5.3)	1 (2.8)	2 (3,6)
	Epigastralgia	1 (5.3)	3 (8.3)	4 (7,3)
	Nausea	1 (5.3)	1 (2.8)	2 (3,6)
Neurological 6 (10.9)	Paresthesia	1 (5.3)	4 (11.1)	5 (9,1)
	Insomnia	-	1 (2.8)	1 (1.8)
Musculoskeletal 2 (3.6)	Arthralgia	1 (5.3)	-	1 (1.8)
	Cramps	-	1 (2.8)	1 (1.8)
Total		19 (100)	36 (100)	55 (100)

 The most prevalent manifestations in men were dermatitis (26.3% of 5/19) and leukopenia (21% of 4/19). In women, dermatitis was observed in 27.8% (10/36) and pruritus in 22.2% (8/36). Occasionally, the manifestations pertained to multiple systems (**Table 1**). 

 In some participants, sweating was recorded in one study and syncope and dizziness in three studies. However, these were not considered possible adverse effects because of the inability to establish a clear causal relationship between the events. 

 Hematological analysis revealed leukopenia in six patients (four men and two women) and thrombocytopenia in three patients (two men and one woman), with the most significant case reaching a platelet count of 46,000/mm^3^. Regarding leukopenia, a decrease between 100 and 3,600 leukocytes was observed in 16 patients, with 14 patients experiencing a relative decrease of > 10% between the leukocyte value before treatment and the value in the second half of the treatment period. 

 Four patients treated with benznidazole discontinued treatment voluntarily because of adverse reactions (three women and one man). The average number of tablets consumed by the patients until the onset of adverse reactions was 61, which is equivalent to approximately 20 days of treatment at a dose of three tablets per day. In women, the average was 51 tablets or approximately 17 days of treatment. In men, the average was 72 tablets or approximately 24 days from the treatment initiation. 

 Bivariate analysis revealed a statistically significant association between female sex and the occurrence of adverse reactions (P = 0.009). Adverse dermatological reactions were more frequently observed among female patients (P = 0.023), whereas adverse hematological reactions were more strongly associated with male sex (P = 0.042) (**Table 2**). 

**Table 2:** Adverse reaction in patients with Chagas disease receiving etiological treatment with benznidazole at the Chagas Disease Outpatient Clinic of the Universidade do Estado do Rio Grande do Norte (ADOC-UERN). Bivariate analysis of the variables sex, any adverse reaction, and dermatological adverse reaction

**Variables**		**Sex**		**X^2^**	**P value**
		**Male n (%)**	**Female n (%)**		
Adverse reaction	Yes	17 (39.5)	26 (60.5)	6.73	0.009^a^
	No	41 (65.1)	22 (34.9)		
Dermatological	Yes	6 (24)	19 (76)	5.203	0.023^a^
	No	10 (58.8)	6 (41.2)		
Gastrointestinal	Yes	3 (42.9)	4 (57.1)	0.081	1^b^
	No	13 (13.3)	22 (21.7)		
Hematological	Yes	6 (75)	2 (25)	5.171	0.042^b^
	No	11 (31.4)	24 (68.6)		
Musculoskeletal	Yes	1 (50)	1 (50)	0.106	1^b^
	No	15 (38.5)	24 (61.5)		
Neurological	Yes	1 (25)	3 (75)	0.321	1^b^
	No	15 (39.5)	23 (60.5)		

a Pearson’s Chi-squared test

b Fisher’s exact test

**Figure 1:**
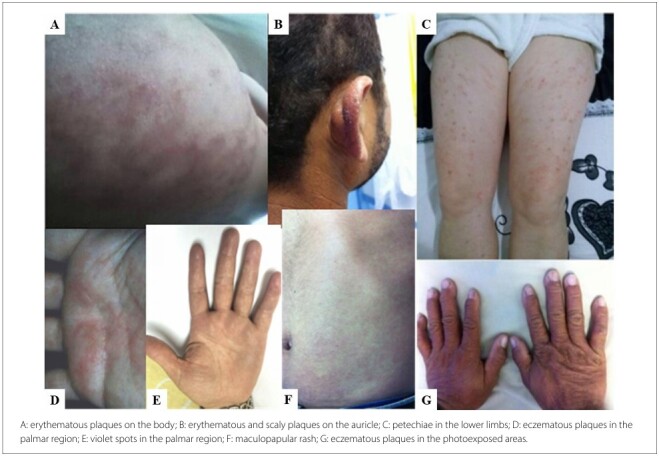
Skin lesions with typical presentations of reactions to benznidazole.

**Figure 1** presents some examples of the dermatological reactions observed in the patients included in this study. 

## DISCUSSION

 Despite the proven efficacy of medications against Chagas disease, reports on the side effects of their use are few. Benznidazole has the best safety and tolerance record, making it the first choice of treatment.^17,18^ The most frequent side effect of benznidazole treatment is dermatitis, which is attributed to hypersensitivity.^19^ In our practice, the majority of treated patients exhibited dermatological conditions as a side effect, with dermatitis and pruritus being prominent. Treatment discontinuation was not required because adverse effects could be managed using corticosteroids and antihistamines, in addition to reducing the dosage twice daily and extending the treatment duration to accommodate the calculated dose for each patient. 

 Leukopenia and thrombocytopenia were the most common hematological reactions. Regarding platelet suppression, the literature reports episodes of thrombocytopenic purpura triggering flushing and the appearance of petechiae in the palmar and plantar regions within minutes of drug ingestion, potentially leading to digestive, urinary, oral, and nasal mucosal hemorrhages.^20^

 Reported as the second most frequent manifestation of side effects,^17,21,22^ gastrointestinal adverse reactions were observed in 14.3% of our patients. Adverse neurological effects were reported after 30 days of treatment, with paresthesia being prominent and more common in women than in men. This finding is similar to those of Pinazo^23^ and Tornheim,^18^ where 27.6% and 29.8% of their patients, respectively, experienced adverse neurological reactions. 

 Among the treated patients, 40.5% experienced adverse reactions, a proportion within the range already described in the literature^17,21,22^ Women showed a higher incidence of side effects than men, supporting the results of previous studies^17,24,25^ The most common categories of adverse reactions in women compared with men were within the dermatological and neurological systems^18,21^ The higher prevalence of side effects in women may be attributed to their greater adherence to treatment and metabolic alterations owing to hormonal levels and corporal composition, as women have less muscle and lower basal metabolism than men.^26^

 A factor that enabled effective follow-up and consequently a low discontinuation rate of etiological treatment was the team’s dedication to providing the best possible care, offering guidance, providing informative materials, and offering the flexibility to adjust symptomatic therapy for patients experiencing adverse reactions. 

## CONCLUSIONS

 This study describes the most frequent adverse reactions associated with the use of benznidazole in patients with Chagas disease. This study highlights the importance of identifying these events to increase the awareness of risks, thereby facilitating professional guidance for patients, instilling greater confidence in treatment, and improving adherence. 

## Data Availability

The data supporting the study findings are available from the corresponding author, José Antonio da Silva Júnior, upon request.
